# Reflecting on 25 years of SPIKES: bad news communication in our modern era

**DOI:** 10.1093/oncolo/oyag064

**Published:** 2026-02-25

**Authors:** Joseph McCollom, Mazie Tsang

**Affiliations:** Department of Palliative and Survivorship Oncology, Parkview Health, Fort Wayne, IN 46845, United States; Division of Hematology/Oncology in the Department of Medicine, Mayo Clinic, Phoenix 85054, Arizona

**Keywords:** palliative care, supportive care, serious illness communication

The turn of the millennium marked a pivotal transition in serious illness communication. At that time, cancer prognosis remained poor, and many clinicians felt pressured to withhold information from patients and their loved ones, fearing that disclosure might diminish hope. Articles published in the late 1990s described clinical conversation but lacked empirical data,^1^ or they assessed patient needs without offering practical guidance.^2^ Guidelines were coalescing around best practices but lacked teachable structures.^3^ In this context, Baile *et al.* introduced the SPIKES protocol in 2000,^4^ a six-step approach to delivering bad news (Figure 1), which remains the most frequently accessed article in *The Oncologist* to date—it has over twice as many citations and an Altmetric score 10 times higher than other articles published that year.

**Figure 1:**
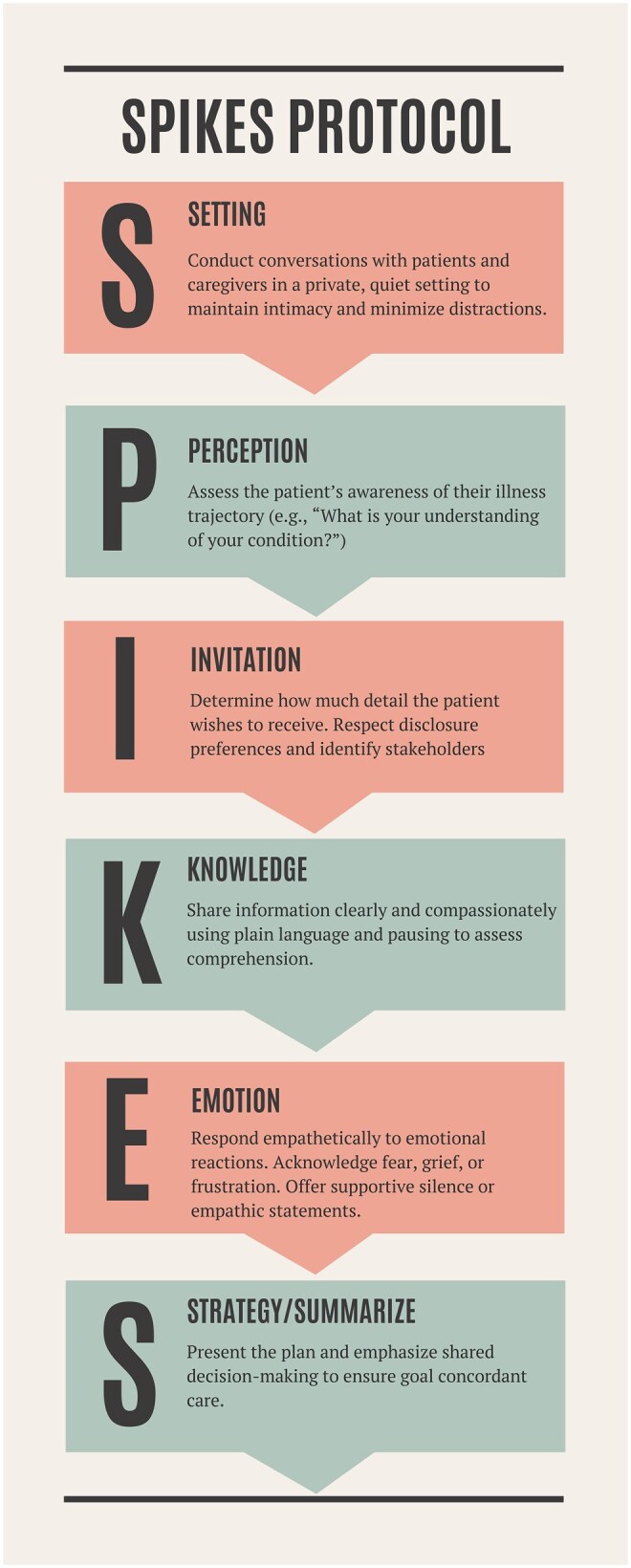
Summary of the six core steps of the SPIKES protocol for compassionate communication.

SPIKES has provided a structured framework for the most challenging conversations in everyday oncology practice, such as disease relapse, new metastasis, treatment toxicity, or end-of-life discussions. This concise protocol has had a profound impact on serious illness communication over the past 25 years, shaping teaching and training, as well as inspiring complementary communication models. Its profound yet straightforward structure is teachable to any medical professional, regardless of background or training. The six components of SPIKES include:

- S: Setting—Conduct conversations with patients and caregivers in a private, quiet setting to maintain intimacy and minimize distractions. Create an intentional space and identify each stakeholder for the patient’s care community. Communicate while sitting down to show intention, and maintain eye contact to enhance presence and show value. Be transparent about the time available for the conversation. Use comforting touch to show connection and a respectful and professional tone. Prepare tissues, and mute all devices.
- P: Perception—Assess the patient’s awareness of their illness trajectory (eg, “What is your understanding of your condition?”) Use open-ended questions to learn more about how the viewpoints of the patient and their invested loved ones align or diverge from the clinical situation. The information obtained will be critical in dispelling misunderstandings, addressing the patient’s wishes, or even acknowledging a denial of serious illness. Tailor the approach to improve accuracy and clinician sensitivity.
- I: Invitation—Determine how much detail patients wish to receive, recognizing that some may desire full disclosure, while others may need information mediated by a trusted individual. Clarifying a patient’s preferences in advance reduces anxiety both for patients and clinicians. Serious illness may change a person’s communication preference—information avoidance may be their coping mechanism. Asking what a patient prefers and offering to answer questions empowers patients in their care.
- K: Knowledge—Share information clearly and compassionately using plain language and pausing to assess comprehension. Signaling to patients that difficult information is coming may ease the emotional impact. Partnering with patients as they process the information will provide comfort. Clinicians should aim to deliver facts in understandable language, avoiding the extremes of overly technical terms or blunt and isolating statements. Each disclosure should be concise, with a clinician check for comprehension. Emphasize that there are always care options, even if cancer-directed therapy is not feasible.
- E: Emotion. Respond empathetically to emotional reactions. Acknowledge fear, grief, or frustration. Offer supportive silence or empathic statements. This may be the most daunting of the six steps, as patient reactions can vary from silence to dramatic outbursts. Name emotions in a respectful way. Acknowledge that their emotions are appropriate given the news. Empathize and partner with patients and their loved ones while leaving space and time to allow them to process their emotions fully. Validating emotions fosters trust and solidarity between clinicians and patients.
- S: Strategy and Summary—Present the care plan and emphasize shared decision-making to ensure goal-concordant care. Clinicians should reinforce a clear plan to alleviate patients’ and caregivers’ anxiety. Clinicians should also address misunderstandings about the purpose and efficacy of treatment and supportive options. Reframing the next steps around a patient’s expressed goals for their care provides clinicians a foundation for ongoing care. By discussing achievable goals, clinicians provide reassurance while ensuring that shared decision-making is patient-centered.

The SPIKES protocol was rapidly integrated into medical education due to its concise and teachable structure. Practical, skills-based workshops enabled learners to practice SPIKES in simulated scenarios. Clinicians trained with SPIKES demonstrated measurable improvements following targeted instruction.^5^ The protocol has been adapted for diverse trainees, including emergency medicine residents,^6^ pharmacy students,^7^ and medical students.^8^ In some instances, the focus of SPIKES has been expanded beyond the patient–clinician relationship to strengthen feedback from instructors to learners.^9^ Importantly, SPIKES laid the foundation for subsequent communication training initiatives. OncoTalk introduced intentional small-group workshops for oncology professionals,^10^ while VitalTalk expanded this model into faculty development programs and bedside coaching, thereby transforming the field of serious illness communication.^11^

Although originally designed for oncology, SPIKES has influenced multiple disciplines. In mental health, for example, the PEWTER (Prepare, Evaluate, Warning, Telling, Emotional Response, Regrouping) model drew on the SPIKES structure,^12^ while the REMAP (Reframe, Expect emotion, Map out patient goals, Align with goals, and Propose a plan) framework expanded the concept from a single disclosure to longitudinal goals-of-care conversations.^13^ Adaptations of the SPIKES format have been published to fit different clinical environments, such as time-pressured emergency departments.^14^ Disclosures have been customized to cultural contexts, including Germany^15^ and the Middle East.^16^ Within oncology, SPIKES has informed treatment failures,^17^ patient-centered decision making,^18^ and oncology nursing.^19^ Adaptations have reworked the SPIKES model for cultural impact^20^ or diagnosis-tailored needs, such as SPIKES-D for dementia care.^21^

In 2015, Walter F. Baile, the lead author on the original SPIKES, revisited SPIKES’ enduring impact in *The Oncologist.^22^* He discussed the model’s influence on patient and clinician communication, from disclosure to the expansion of emotional intelligence among oncology professionals. Baile reflected that conceptualizing the cancer experience as “a war” and death as “defeat” has made delivering bad news in this field even more challenging.^22^ He acknowledges the expansion of the SPIKES protocol to address stress management, anticipatory emotional experiences, and cultural sensitivity. He emphasizes the increasing significance of mindfulness practices and role play in enhancing communication training for serious illness conversations.^22^

As digital transformation—from telehealth to artificial intelligence (AI)—reshapes the landscape of serious illness communication, the SPIKES protocol remains an important guide for clinicians navigating these technological shifts while preserving human connection and communication of sensitive information. The COVID pandemic accelerated the widespread adoption of telehealth, requiring many oncology professionals to adapt face-to-face conversations to virtual platforms.^23^ Although virtual interviews might make patients feel more comfortable when they are at home, they can lead to more distractions in public settings and a decreased ability to interpret nonverbal cues or include off-screen family members. Beyond telehealth, the rise of AI and large language models has led to exploration of their role in improving clinician communication and patient care. For example, “virtual patient” simulations have helped emergency medicine physicians and residents break bad news,^24^^,^^25^ and predictive AI algorithms may identify patient outcomes or distress^26^—yet the human connection fostered by SPIKES remains irreplaceable. Another digital challenge is electronic health record transparency, which sometimes leads patients to learn difficult news before meeting with their oncologist. The SPIKES protocol can help clinicians prepare for these scenarios, ensuring that patients receive guidance before attempting to navigate in isolation. This framework ensures that conversations remain patient-centered despite technological shifts. The efforts to incorporate SPIKES into modern practice require intentional patient education that is tailored to their personal preferences.^27^  Table 1 outlines strategies to adapt SPIKES to our digital healthcare environment. Although these digital revolutions have transformed how serious news is communicated, SPIKES endures as a foundation for compassionate communication in an evolving healthcare landscape.

**Table 1:** Adapting the SPIKES protocol to an evolving digital healthcare delivery landscape.

SPIKES protocol steps	Guidance	Technology considerations
**S—Setting**	Ensure privacy and comfort whether fully virtual, in-person, or hybrid disclosure settings.	For telehealth encounters, ensure technology is updated and conducted on charged devices with adequate network access. For hybrid visits, ensure loved ones have access to links in advance of the encounter. Ensure adequate camera placement, encryption, and sound quality with backup headphones for all participants.
	Minimize interruptions by being proactive in preventing alerts and silencing devices when possible.	Clinicians should turn off push notifications or set privacy mode on devices, as well as encourage other participants to do the same. When possible, diverting critical medical communication to a delegate or community to covering partners can reduce disruptions.
**P—Perception**	Use open-ended questions to understand what patients perceive from online sources.	Patients have wide access to medical information, but many online sources may not draw from credible evidence-based literature. Acknowledge a patient’s desire for partnership as they research their condition and treatment options while offering reputable sources.
	Understand potential online misinformation from social media or AI hallucinations.	Unproven wellness interventions or alternative therapies can be rapidly disseminated via social media. Well-meaning patients or loved ones may bring unsubstantiated data hallucinated by Large Language Models when entering their information into a prompt. Clinicians should affirm and partner with patients while emphasizing high-quality evidence.
**I—Invitation**	Ask how patients wish to receive their results via the online portal.	Electronic Health Records (EHR) can auto-release results to patients. Educate patients on how to set their communication preferences on the online portal. Encourage patients to have a portal designee if they feel anxious about or distressed by results.
	Offer to harness virtual communication to allow distant loved ones to be engaged in the encounter.	Video conferencing can create opportunities to involve family members who may be separated by physical distances. Assist patients in securing reliable technology to facilitate these discussions. Clinicians should obtain consent prior to reaching out for virtual connections.
**K—Knowledge**	Discuss precision genomics testing with a focused approach.	Extensive sequencing results could overwhelm patients with complex and technical information. Consider focusing on driver mutations that have a direct correlation to a patient’s prognosis, treatments, or clinical trial options.
	Tailor information presentation to a patient’s health literacy. Portable technology like laptops or tablets could provide helpful visual aids.	The flexibility of digital devices may be able to bring comprehensive graphics or videos that can augment a clinician’s communication of serious illness. Generative AI may be able to create professional and helpful infographics, but they should be verified for accuracy.
**E—Emotion/Empathy**	Usie visual and verbal cues to acknowledge emotions on virtual platforms.	Hybrid and telehealth interactions may lose the nonverbal cues to emotional expression. Intentional pauses and expressive body movements can augment emotional acknowledgement on a virtual platform.
**S—Strategy/Summary**	Harness electronic patient-reported outcome measures to gauge the longitudinal impact of cancer-directed management.	Online portals and 3rd party applications may have the ability for the patient to communicate in real time what they are experiencing while on treatment. This seamless communication or use of electronic patient-reported outcome assessments could allow for consistent access to clinicians between appointments.

The lasting strength of SPIKES lies in its adaptability. Over the past 25 years, it has transformed serious illness communication in oncology, grounding these difficult conversations in intentionality, compassion, and structure. As healthcare delivery continues to evolve, SPIKES remains an indispensable cornerstone for a compassionate and patient-centered approach that must always be at the heart of serious illness conversations.

## Data Availability

The data underlying this article are available in the article and in its online supplementary material.
